# Sarcopenia and chemotherapy-mediated toxicity

**DOI:** 10.1590/S1679-45082016MD3740

**Published:** 2016

**Authors:** Maria Cecília Monteiro Dela Vega, Alessandro Laviano, Gustavo Duarte Pimentel

**Affiliations:** 1 Centro Brasileiro de Radioterapia, Oncologia e Mastologia, Goiânia, GO, Brazil.; 2 Department of Clinical Medicine, Sapienza University, Rome, Italy.; 3 Laboratório de Investigação em Nutrição Clínica e Esportiva, Faculdade de Nutrição, Universidade Federal de Goiás, Goiânia, GO, Brazil.

**Keywords:** Sarcopenia/drug therapy, Muscles/pathology, Neoplasms, Inflammation

## Abstract

This narrative review focuses on the role of sarcopenia and chemotherapy-induced toxicity in cancer patients. Consistent evidence shows that sarcopenia in cancer patients leads to decreased overall survival by influencing treatment discontinuation and dose reduction. Therefore, sarcopenia should be considered a robust prognostic factor of negative outcome as well as a determinant of increased healthcare costs.

## RESUMO

Esta revisão narrativa descreve o papel da sarcopenia e a toxicidade mediada pela quimioterapia em pacientes com câncer. Diversas evidências consistentes mostram que a sarcopenia em pacientes com câncer induz à menor sobrevida global, por influenciar na interrupção do tratamento e na redução da dose. Portanto, a sarcopenia pode ser considerada um importante fator de prognóstico de desfecho negativo, além de um determinante de maiores custos em saúde.

## INTRODUCTION

**Figure 1:**
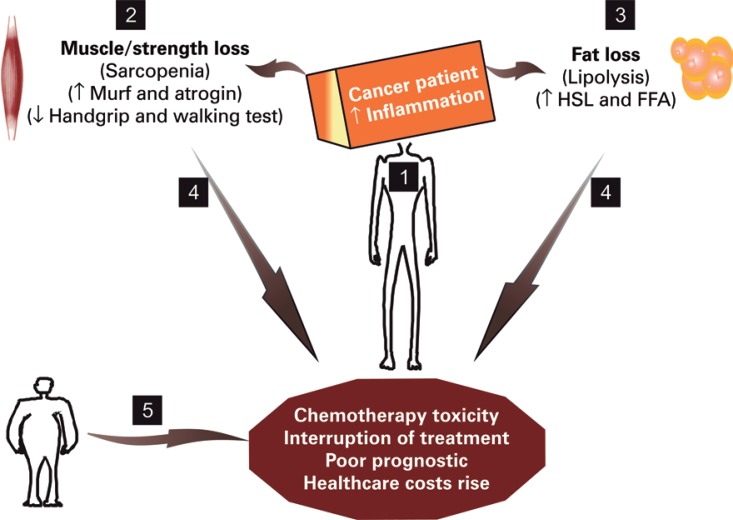
Cancer sarcopenic patient and mechanisms that underlying the chemotherapy-mediated toxicity. Cancer individuals are affected by high-grade inflammation derived from tumor that leads to fat and muscle loss (1). The mechanisms associate with sarcopenia involves the overexpression of the muscle RING finger-containing protein 1 (MURF-1) and atrogin in the skeletal muscle (2) and an explanation of lipolysis occurs by hormone-sensitive lipase activation and free fatty acids secretion (3). Taken together, cancer subjects classified with sarcopenia and adipose tissue atrophy have increased chemotherapy toxicity, reduced cycles or interruption treatment, and poor prognostic (4). In addition, sarcopenia induces chemotherapy-mediated toxicity in cachectic patient as well as in cancer obese-sarcopenic individuals. However, the underlying mechanisms are not clear yet (5). MAFbx/atrogin: muscle atrophy F box protein

HSL: hormone-sensitive lipase; FFA: free fatty acids.

Sarcopenia is the major feature of cancer cachexia, and is associated with reduced quality of life and survival.^(1,2)^ It is defined as low skeletal muscle mass, grip strength and gait speed.^(3)^ There are several reasons for muscle mass depletion in cancer patients, such as higher energy expenditure, anorexia, inflammation and unbalanced cancer metabolism.^(1,4,5)^ Some evidences suggest that tumor mass is responsible for: (1) production of inflammatory cytokines (*e.g*. tumor necrosis factor and interleukin-1), which leads to release of numerous sarcopenia-related myofibrillar proteins, for *e.g.* activin A acts on its receptor, activating the SMADs 2/3, inhibiting the AKT/mTOR signaling, and leading to muscle atrophy; at the same time, the activation of the muscle RING finger-containing protein 1 (MURF-1) and the muscle atrophy F box protein (MAFbx/atrogin) occurs, potentiating skeletal muscle and strength and gait speed loss and consequently of high frailty; (2) inflammatory molecules that diminish the appetite; and (3) lipolytic mediators that induce lipolysis of adipose tissue via activation of adrenergic pathways with stimulation of protein kinase A, hormone sensitive lipase (HSL) and adipose triglyceride lipase (ATGL), releasing free fatty acids.^(5-9)^ In addition, these etiologic factors of sarcopenia in cancer are also observed in oncological therapies, such as surgery, chemotherapy and radiotherapy, which cause nausea, vomiting, loss of taste, fatigue and pain. Taken together, the combined deleterious factors on body composition with oncological treatments links adipose-muscle-hypothalamic tissue axis, potentiating sarcopenia and malnutrition in cancer patients. Figure 1 illustrates these findings.

In cancer cachexia and in obese subjects, sarcopenia can be defined using an appendicular lean soft tissue index when quantified by dual-energy X-ray absorptiometry (DEXA) below 7.26kg/m^2^ for men and 5.45kg/m^2^ for women.^(1,3)^Although sarcopenia outcomes involve differences between the sexes and its characterization is related mainly to DEXA, cancer patients are frequently diagnosed and followed up by computerized tomography (CT), after chemotherapy.^(10-13)^ Computerized tomography in sarcopenic cancer patients can be used taking into account not only changes in muscle and adipose tissues, as well as tumor masses, but also according to age, race, tumor site and cancer status. In this way, choosing only one method to assess and treat our patients can provide summarized and timely pieces of information on anthropometric and tumor-related measurements. Although the criteria for classifying sarcopenia in cancer patients are not yet totally clear,^(1)^ Computerized tomography is used to measure the lumbar (L3) level muscle mass and the cutoff points of ≤38.5cm^2^/m^2^ in women, and ≤52.4cm^2^/m^2^ in men, has been adopted to define sarcopenia.^(12)^

It is known that sarcopenic cancer patients have reduced overall survival.^(14)^ For example, data from cancer individuals show high sarcopenia prevalence, with a range of 21 to 71% for both women and men.^(13-19)^ Moreover, neoadjuvant chemotherapy in oncological patients can increase the prevalence of sarcopenia by 17% up to the end of treatment.^(20)^ In addition, sarcopenia causes higher grade toxicity in metastatic renal cancer patients, which may lead to dose-limiting toxicity already in the first cycle, in approximately 30% of subjects.^(21)^ Based on high prevalence and healthcare expenditure with sarcopenia treatment, we sought to review the etiological and pathophysiological aspects, prevalence and costs of sarcopenia in cancer subjects.

To achieve our goal, we adopted a narrative literature review and included the clinical studies published in English in the database PubMed MEDLINE^®^. The keywords used were “sarcopenia”, “cancer”, “inflammation”, “strength” and “lipolysis”.

Healthcare costs of sarcopenia in older American communities represent approximately 1.5% of total healthcare expenditure for a year, with an estimated expense of US$ 10.8 billion for men, and US$ 7.7 billion for women, in 2000.^(22)^ These costs and the consequences of skeletal muscle mass depletion, such as increased physical and functional disability, are estimated for around the world mainly in the elderly and people with inflammatory diseases.

Increased C-reactive protein (CRP) concentrations and accelerated muscle mass loss during chemotherapy cycles have been recently observed in patients with advanced cancer, such as colorectal, biliary and upper gastrointestinal tracts. Likewise, the prevalence of elevated CRP levels increases by approximately 10%, from the first to the last chemotherapy cycles.^(23)^ Additionally, palliative chemotherapy for non-small cell lung cancer patients, in regimes of chemotherapy with carboplatin, vinorelbine and gemcitabine, is also a risk factor for sarcopenia. A reduction in muscle mass by 1.4kg after 9 weeks of chemotherapy has already been described.^(18)^

A cross-sectional study performed with stage III colon cancer subjects, on adjuvant chemotherapy (oxaliplatin, 5-fluorouracil and leucovorin) revealed that the low muscle mass at baseline was associated with increased occurrence (67%) of all grade 3-4 chemotherapy-induced toxicities. The same result was observed after adjusting for age, sex, hemoglobin levels, and glomerular filtration rate.^(24)^ Although the diagnosis of sarcopenia in patients with colon, lung, esophagogastric and other types of cancer^(18,24-27)^ did not demonstrate low overall survival, even with chemotherapy, there was increased mortality, poor prognostic factors and shorter progression-free survival.^(27,28)^

When comparing obese cancer patients with malnutrition, esophageal cancer patients undergoing neoadjuvant chemotherapy (cisplatin and 5-fluorouracil), we verified that sarcopenic-eutrophic and sarcopenic-obese subjects had an odds ratio of 2.4 and 5.5 times higher, respectively, for dose-limiting toxicity than non-sarcopenic individuals. This indicates that muscle mass measurement can represent a determining factor when dosing chemotherapy, mainly in subjects with increased adiposity and decreased muscle mass.^(17)^ Likewise, in esophagogastric cancer subjects being treated with cisplatin, 5-fluorouracil, epirubicin and capecitabine, we observed that sarcopenia increased the risk of dose-related toxicity threefold.^(25)^ Furthermore, sarcopenia is associated with increased risk of severe postoperative abnormalities in colorectal peritoneal carcinomatosis during the intraperitoneal chemotherapy.^(13)^

Based on dose-limiting toxicity, sarcopenic patients with breast cancer, treated with capecitabine, present with higher grade toxicity than non-sarcopenic individuals.^(29)^

In advanced non-small cell lung cancer individuals, aggravated chemotherapy-induced anorexia and an association between skeletal muscle mass loss with disease and treatment prognostic factors were observed.^(30)^ In metastatic colorectal cancer patients only sarcopenia is linked to toxicity, which corresponded to approximately 38% of subjects.^(19)^

Although studies report that anorexia and cachexia are major contributors to the adverse effects of chemotherapy, this review reported that over a few years there have been several evidences showing that sarcopenia potentiates chemotherapy-induced toxicity, and reduces overall survival in cancer patients submitted to oncologic therapy. Furthermore, it is interesting to consider that sarcopenia is not exclusive in patients receiving chemotherapy, but it also occurs in those on radiation therapy, which causes weight loss.^(31)^

Additionally, the dose for most chemotherapeutical treatments is based on the body surface area.^(32)^ However there is growing evidence suggesting this technique, and its associated risks of toxicity, fails particularly in sarcopenic cancer individuals, independently of the body mass index.^(13,18,24,32-34)^ Although it was suggested, in 2013, that the chemotherapy dose in obese cancer patients should be calculated on the body surface area, taking into count the patient’s weight,^(35)^ it is important that quantification of skeletal muscle mass by appendicular lean soft tissue index via DEXA or CT be used at diagnostic examination, in order to calculate a tolerable chemotherapy dose.

Likewise, there are evidences indicating that muscle mass depletion is associated with the discontinuation of chemotherapy and dose reduction.^(21,36,37)^ On the other hand, it is known that a 10% lessening in sarcopenia prevalence could save approximately US$ 1.1 billion per year with healthcare-related economic costs, in the United States.^(22)^

In addition to muscle mass, handgrip strength has been recognized in clinical practice to evaluate fragility and muscle function, monitor body composition and assist in making prognosis of cancer.^(38)^Thus, future studies may adopt this strategy as a simple tool to keep up with cancer patient features with sarcopenia.

Therefore, these conclusions can be drawn from these data: (1) cancer cachexia patients are susceptible to sarcopenia before and after chemotherapy; (2) skeletal muscle mass loss occurs during chemotherapy cycles leading to a poorer prognosis; (3) sarcopenia is recognized as a metabolic abnormality with elevated healthcare costs; (4) sarcopenia worsens chemotherapy-mediated toxicity in sarcopenic obese, eutrophic and malnourished subjects; (5) muscle mass attenuation is implicated in the reduction of chemotherapy cycles and dosage, which hinders positive outcomes; (6) In the absence of a tool to identify sarcopenia, at least in part, handgrip strength can be used to control the prognostic factors of cancer subjects, once this approach is faster and cheaper than other anthropometric methods. Figure 1 summarizes the possible findings regarding sarcopenia as a risk factor for chemotherapy-mediated toxicity.

Skeletal muscle mass loss is a risk factor for chemotherapy-mediated abnormalities, mainly with regard to toxicity and low response to both oncologic and nutritional treatments.
